# Sex-stratified trends in hypoplastic left heart syndrome-related mortality among children and young adults in the United States, 1999–2024: a pre- and post-COVID-19 analysis

**DOI:** 10.3389/fped.2026.1890070

**Published:** 2026-08-04

**Authors:** Akshaya Srinivasan, Aarifa Banu, Soumiya Nadar, Cheryl Lewis, Taha Kassim Dohadwala, Ashwin Bijoy, Carol Lewis, Abna Abdulla, Sakina Mohamadally Ladha, Thisuri Mendis, Angeline Abraham

**Affiliations:** 1 Department of Medicine, Tbilisi State Medical University, Tbilisi, Georgia; 2 Department of Medicine, David Tvildiani Medical University, Tbilisi, Georgia; 3 Department of Medicine, Alte University, Tbilisi, Georgia; 4 Department of Medicine, School of Medicine, Queen’s University Belfast, Belfast, United Kingdom

**Keywords:** CDC WONDER, congenital heart disease mortality, COVID-19 pandemic impact, hypoplastic left heart syndrome, sex-stratified mortality trends

## Abstract

**Background:**

Hypoplastic left heart syndrome (HLHS) is a congenital heart disease that accounts for approximately 3% of congenital heart defects, making it a leading cause of mortality in this population. Timely staged palliation, such as Norwood, Glenn, and Fontan, are required to reduce the incidence of adverse outcomes. Data over the long term suggest that only about 31% of patients with HLHS are alive at age 35 years after Fontan completion. However, national mortality trend data stratified by sex, extending through 2024 and spanning the COVID-19 era, have not been characterised.

**Methods:**

Data for this study were obtained from the CDC WONDER (Centres for Disease Control and Prevention Wide-ranging Online Data for Epidemiologic Research) Multiple Cause of Death database using ICD-10 code Q23.4 from 1999 through 2024 in individuals aged 0–24 years. Joinpoint regression was used to analyse trends. Analyses were conducted using a Poisson variation model with Monte Carlo permutation testing. Statistical significance was defined as *p* < 0.05 based on two-tailed testing and was stratified by sex.

**Results:**

A total of 10,138 deaths were identified in the United States during the study period. Among these, 4,285 were females, and 5,853 were males. Joinpoint regression identified a declining trend from 1999 to 2021 (APC: −2.22%; 95% CI: −3.08 to −1.89; *p* = 0.006), with a non-significant increase from 2021 to 2024 (APC: +4.97%; *p* = 0.156). In 2020, total HLHS-related deaths reached their lowest point (*n* = 287) before rising consecutively to 358 in 2024. Female mortality trends declined throughout the study period (APC: −1.96%; 95% CI: −2.61 to −1.33; *p* < 0.001) compared with males, who remained consistently high.

**Conclusion:**

HLHS mortality has declined following surgical advances and perioperative care management. Persistent higher mortality rates were noted among males than females throughout both pre- and post-COVID eras. The rise in post-pandemic mortality is a new and concerning trend that warrants close national monitoring. These trends emphasise the need for uninterrupted staged palliation and continued population-level surveillance of HLHS outcomes.

## 1 Introduction

Hypoplastic Left Heart Syndrome (HLHS) is one of the most severe and life-threatening forms of congenital heart defects, characterised by severe underdevelopment of left-sided cardiac structures. Neonates born with HLHS require a patent ductus arteriosus to survive, as cardiovascular collapse and mortality are otherwise near-certain (1). HLHS is a rare condition, occurring in approximately 1 in 5,000 live births and accounting for approximately 3% of all congenital heart disease cases (2). Without immediate surgical intervention, HLHS is universally fatal.

The management of HLHS follows a three-stage surgical pathway: the Norwood procedure (Stage 1), the bidirectional Glenn shunt (Stage 2), and Fontan completion (Stage 3) (3). Despite meaningful advances in neonatal cardiac surgery, long-term outcomes remain challenging. Current data show that only approximately 31% of patients survive to 35 years of age following Fontan palliation (4). The Norwood procedure is associated with in-hospital survival exceeding 90% in high-volume hospitals (3). Still, a 30-year series from single institutions, comprising 1,663 Norwood procedures, reported a mortality of 25.9% across the full study period, which later improved to 11.2%–15.7% in the most recent studies (5). In multicentre series, interstage mortality between Norwood and Glenn procedures has been reported to range from 2% to 20% (6). In the Pediatric Heart Network's Single Ventricle Reconstruction (SVR) trial, transplant-free survival was about 60% in 6 years, in the full randomised cohort (7)^,^ and decreased to 59% (right ventricle-to-pulmonary artery shunt group) and 54% (modified Blalock–Taussig–Thomas shunt group) by 12 years of age (8). Surviving adults continue to suffer significant morbidities, including heart failure, arrhythmias, and complications arising from the unphysiological nature of Fontan circulation (4, 9). High-volume centres now report hospital survival rates exceeding 90% for the Norwood procedure (3), and one-year survival in Texas improved from 48.7% (1999–2004) to 64.8% (2014–2017) (10). Nevertheless, 35-year transplant-free survival remains approximately 31%, underscoring that HLHS is a lifelong condition requiring specialised, uninterrupted care (11).

National mortality surveillance remains critical for informing clinical practice and policy. Previous analyses using CDC WONDER data have relied on broader ICD-10 codes (Q20–Q26) without isolating HLHS specifically (12). The most recent HLHS-specific analysis using CDC WONDER was limited to children under 15 years of age and ended in 2020, using simple year-to-year percentage change (13). That analysis did not capture COVID-era disruptions, post-pandemic trends, or outcomes in older adolescents and young adults aged 15–24 years. While prior investigations have identified persistent racial and ethnic disparities in cardiac outcomes (14, 30), no study to date has provided nationwide joinpoint regression-based trend analysis extending to 2024, stratified by sex. Therefore, this study aimed to characterise national trends in HLHS-related mortality among individuals aged 0–24 years in the United States from 1999 to 2024, stratified by sex, using CDC WONDER data and Joinpoint regression analysis.

## 2 Methods

### 2.1 Data source

Mortality data were obtained from the Centers for Disease Control and Prevention's Wide-ranging Online Data for Epidemiologic Research (CDC WONDER) Multiple Cause of Death Public Use database for the years 1999 through 2024 (15). HLHS-related deaths were identified using the International Classification of Diseases, Tenth Revision (ICD-10) code Q23.4 as the underlying cause of death. The study period was defined as 1999 through 2024 because 1999 is the year the United States established ICD-10 coding for mortality data, and CDC WONDER does not provide ICD-10-coded mortality data before this year; therefore, 2024 represents the most recent year for which the finalised and preliminary mortality data were available from CDC WONDER during the time of analysis. The analysis included individuals aged 0–24 years across four age groups: less than 1 year, 1–4 years, 5–14 years, and 15–24 years. As this study relied solely on publicly available, de-identified aggregate data, institutional review board approval was not required.

### 2.2 Outcome measure

The primary outcome was the age-adjusted mortality rate (AAMR) per 100,000 population, standardised to the 2000 United States Standard Population. Crude mortality rates (CMR) were additionally calculated for descriptive purposes. All rates are expressed per 100,000 persons.

### 2.3 Stratification

Mortality data were stratified by calendar year (1999–2024) and sex (male and female). Race and ethnicity stratification was attempted; however, data for Asian or Pacific Islander and American Indian or Alaska Native subgroups were subject to suppression by CDC WONDER in multiple study years due to cell counts falling below the reportable threshold of ten deaths, precluding reliable trend analysis for these groups. This represents an acknowledged limitation of the present study.

### 2.4 Statistical analysis

Temporal trends in AAMR were assessed using the Joinpoint Regression Program version 5.0.2 (National Cancer Institute, Bethesda, MD). This program fits log-linear regression models to identify statistically significant changes in trend direction and magnitude over time, using the Monte Carlo permutation test to determine the optimal number of joinpoints. A Poisson variation model was specified, with a maximum of 4 joinpoints permitted per analysis. The annual per cent change (APC) with corresponding 95% confidence intervals (CI), were calculated for each identified trend segment. A trend was considered statistically significant when the 95% CI for the APC did not include zero. Analyses were performed separately for the overall population, males, and females. Statistical significance was defined as *p* < 0.05 using two-tailed testing. The pre-COVID era was defined as 1999–2019, and the post-COVID era as 2020–2024.

## 3 Results

### 3.1 Overall mortality trends

From 1999 to 2024, 10,138 deaths occurred in the United States among persons aged 0 to 24 years with HLHS as the underlying cause of death, including 5,853 males and 4,285 females. CDC WONDER is a mortality surveillance database and therefore does not collect the total number of individuals in the United States living with HLHS; consequently, the 10,138 deaths identified in this study cannot be expressed as a proportion of the total population living with HLHS. For context, with an estimated birth prevalence of approximately 1 in 5,000 live births and around 3.6–4.0 million annual US births over the study period, the expected number of incident HLHS cases nationally would be 18,000–20,000 over these 26 years. Therefore, the 10,138 deaths identified here represent a substantial proportion, though this comparison should be interpreted cautiously given the differing data sources and the approximate nature of birth prevalence estimates. Overall HLHS-related mortality decreased significantly from 1999 to 2021 (APC: −2.22%; 95% CI: −3.08 to −1.89; *p* = 0.006). However, this downward trend reversed to a non-significant increase from 2021 to 2024 (APC: +4.97%; 95% CI: −1.33 to 13.69; *p* = 0.156). Joinpoint regression identified one change in trend in 2021. HLHS-related deaths reached the lowest recorded level (*n* = 287) in 2020, with mortality rising from 290 deaths in 2021 to 358 deaths in 2024, representing a 24.7% increase from the pandemic nadir.

### 3.2 Sex-stratified trends

The number of males who died due to HLHS exceeded that of females throughout the study period (5,853 vs. 4,285). Crude mortality rates among males exceeded those of females at all time points, with the male-to-female mortality ratio varying between 1.20 and 1.62. Joinpoint analysis identified a trend change for males in 2021 (95% CI: 2001–2022), splitting the data into two periods. Male mortality did not decrease significantly from 1999 to 2021 (APC: −2.17%; 95% CI: −6.04 to 7.14; *p* = 0.084). After 2021, there was a non-significant reversal of trend (APC: +4.95%; 95% CI: −2.59 to 15.06; *p* = 0.492). No joinpoint was identified among females, and the female death rate declined significantly throughout the entire study period (APC: −1.96%; 95% CI: −2.61 to −1.33; *p* < 0.001).

**Figure 1:**
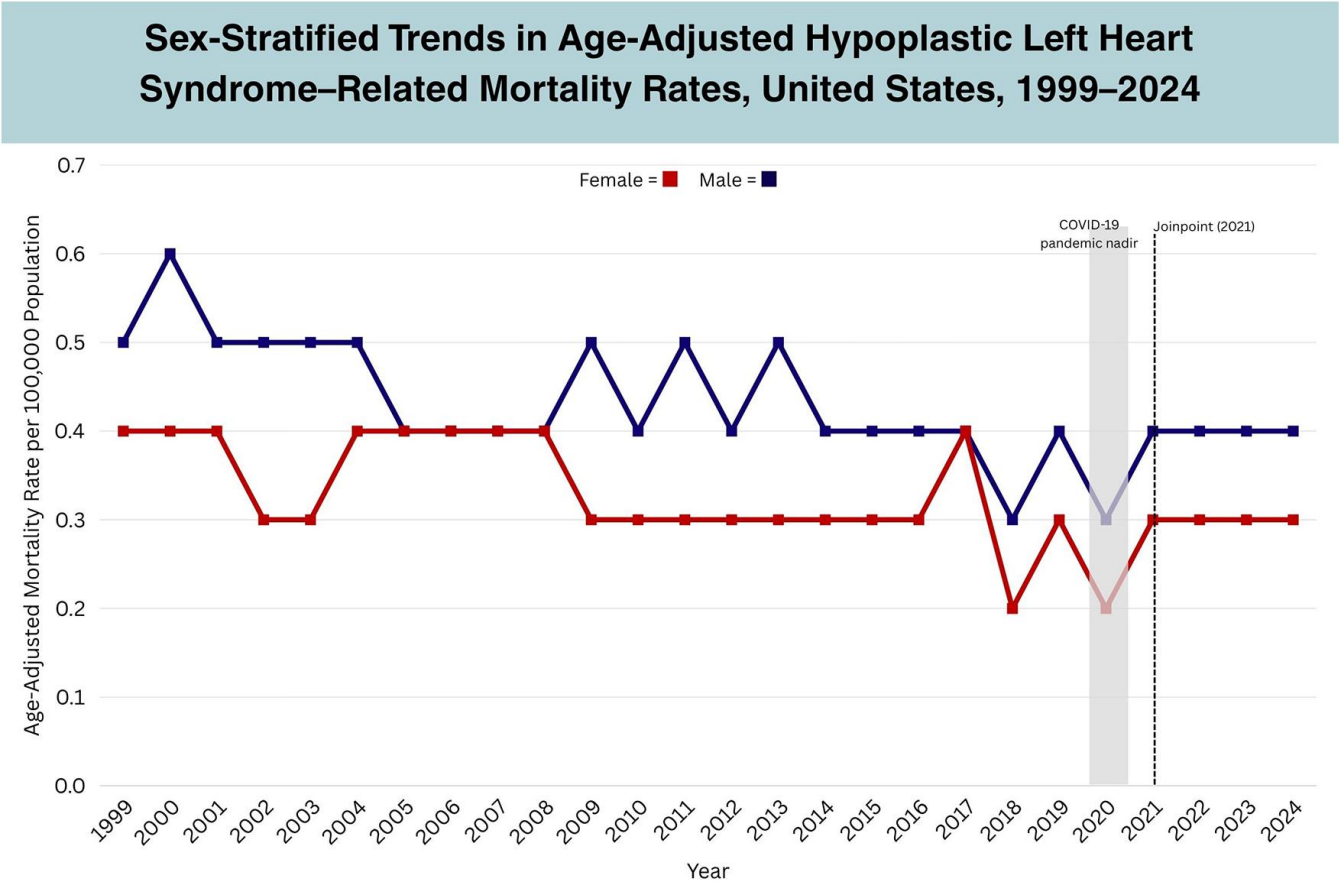
Trends in age-adjusted HLHS-related mortality rates by sex, United States, 1999–2024. The shaded area represents 2020 (COVID-19 pandemic nadir, *n* = 287). The dashed vertical line indicates the joinpoint identified at 2021. Year-to-year fluctuations reflect rounding of age-adjusted rates to one decimal place as reported by CDC WONDER. AAMR, age-adjusted mortality rate per 100,000 population, standardized to the 2000 US Standard Population; HLHS, hypoplastic left heart syndrome; CDC WONDER, Centers for Disease Control and Prevention Wide-ranging Online Data for Epidemiologic Research.

Both males and females demonstrated a reduction in deaths in 2020, followed by a gradual increase through 2024, consistent with the overall post-COVID trend. Detailed annual data and Crude mortality rates are presented in Table 1. Joinpoint regression results are summarised in Table 2. Trends in age-adjusted mortality rates by sex from 1999 to 2024 are illustrated in Figure 1.

**Table 1:** Annual HLHS-related deaths and age-adjusted mortality rates and crude mortality rate by sex, United States, 1999–2024.

Year	Female				Male				Total Deaths
Year	Deaths	Population^a^	AAMR	CMR	Deaths	Population	AAMR	CMR	Total Deaths
1999	213	48,114,203	0.40	0.44	274	50,517,196	0.50	0.54	487
2000	217	48,504,241	0.40	0.45	295	50,933,025	0.60	0.58	512
2001	193	49,093,055	0.40	0.39	285	51,570,772	0.50	0.55	478
2002	179	49,466,931	0.30	0.36	266	51,950,234	0.50	0.51	445
2003	172	49,785,739	0.30	0.35	261	52,231,765	0.50	0.50	433
2004	197	50,055,762	0.40	0.39	236	52,544,152	0.50	0.45	433
2005	193	50,221,682	0.40	0.38	242	52,743,533	0.40	0.46	435
2006	182	50,404,205	0.40	0.36	224	52,956,348	0.40	0.42	406
2007	188	50,652,523	0.40	0.37	244	53,174,907	0.40	0.46	432
2008	186	50,915,859	0.40	0.37	227	53,383,017	0.40	0.43	413
2009	162	51,129,889	0.30	0.32	237	53,534,624	0.50	0.44	399
2010	152	51,246,786	0.30	0.30	214	53,606,769	0.40	0.40	366
2011	150	51,296,618	0.30	0.29	240	53,702,363	0.50	0.45	390
2012	173	51,333,144	0.30	0.34	214	53,754,859	0.40	0.40	387
2013	148	51,304,455	0.30	0.29	232	53,739,070	0.50	0.43	380
2014	155	51,338,950	0.30	0.30	218	53,708,826	0.40	0.41	373
2015	164	51,242,161	0.30	0.32	224	53,622,842	0.40	0.42	388
2016	154	51,057,677	0.30	0.30	216	53,428,419	0.40	0.40	370
2017	173	50,953,033	0.40	0.34	207	53,318,814	0.40	0.39	380
2018	132	50,772,757	0.20	0.26	166	53,083,487	0.30	0.31	298
2019	125	50,497,767	0.30	0.25	203	52,760,589	0.40	0.38	328
2020	123	50,318,789	0.20	0.24	164	52,530,321	0.30	0.31	287
2021	125	50,670,386	0.30	0.25	165	52,984,947	0.40	0.31	290
2022	136	50,686,033	0.30	0.27	198	53,092,925	0.40	0.37	334
2023	142	50,529,650	0.30	0.28	194	52,855,483	0.40	0.37	336
2024	151	51,060,484	0.30	0.30	207	53,435,417	0.40	0.39	358
Total	4,285	—	—		5,853	—	—		10,138

AAMR, age-adjusted mortality rate per 100,000 population, standardised to the 2000 US Standard Population; CMR, crude mortality rate per 100,000 population (deaths/sex-specific U.S. population aged 0–24 years × 100,000), calculated directly from CDC WONDER death and population counts; HLHS, hypoplastic left heart syndrome; CDC WONDER, Centers for Disease Control and Prevention Wide-ranging Online Data for Epidemiologic Research; ICD-10 code Q23.4.

Blue shading=2020 (COVID-19 pandemic nadir, *n* = 287). Yellow shading=2021 (joinpoint identified by regression analysis).

a Population=U.S. Census Bureau estimate of the total sex-specific population aged 0–24 years in each calendar year (i.e., the general U.S. population at risk, not the number of individuals with HLHS), as provided by CDC WONDER and used as the denominator for rate calculation.

**Table 2:** Joinpoint regression results for HLHS-related mortality by sex, United States, 1999–2024.

Group	Segment	Period	APC (%)	95% CI	*p*-value	Statistically significant
Overall	1	1999–2021	−2.22	−3.08 to −1.89	0.006	Yes^a^
Overall	2	2021–2024	+4.97	−1.33 to 13.69	0.156	No
Male	1	1999–2021	−2.17	−6.04 to 7.14	0.084	No
Male	2	2021–2024	+4.95	−2.59 to 15.06	0.492	No
Female	1	1999–2024	−1.96	−2.61 to −1.33	<0.001	Yes^a^

APC, annual percent change; CI, confidence interval.

Joinpoint identified at 2021 for the Overall and Male groups (95% CI: 2001–2022 for Male; 2008–2022 for Overall). No joinpoint identified for the Female group.

Analysis performed using Joinpoint Regression Program version 5.0.2 (National Cancer Institute).

Poisson variation model with Monte Carlo permutation testing.

a Statistically significant (95% CI does not cross zero).

## 4 Discussion

### 4.1 Overall AAMR decline

The age-adjusted mortality rate (AAMR) for HLHS declined significantly between 1999 and 2021 in both males and females. This decline reflects improvements in surgical and perioperative care. The evolution of the Norwood procedure has been critical, and high-volume centres now report hospital survival rates exceeding 90% (3). Meta-analytic data demonstrate progressive improvements in early survival after Stage I palliation over time (16). Enhancements in Stage I palliation, particularly the implementation of the right ventricle-to-pulmonary artery (RVPA) conduit instead of the modified Blalock–Taussig shunt, have improved early outcomes, though long-term survival does not differ substantially between the two techniques (8). Meta-analyses favour the Norwood strategy, as hybrid palliation is associated with increased early and interstage mortality and increased reintervention (17, 18). This has resulted in long-term survival benefit, with increasing numbers of patients with HLHS surviving into adolescence and young adulthood (11, 19). However, the rate of mortality decline has slowed in recent years, likely reflecting diminishing returns from incremental surgical refinement, an increasing cumulative comorbidity burden, and a shift in mortality toward older age groups (20).

### 4.2 Sex disparity

Age-adjusted mortality rates for HLHS were consistently higher in males than in females throughout the 26-year study period. The observed sex difference in HLHS-related mortality is likely multifactorial and cannot be attributed to a single cause. Several factors contribute to it. The increased male AAMR reflects the increased incidence/prevalence of HLHS among males. In most population-based studies, severe congenital heart defects, including HLHS, clearly show an increased male incidence, which suggests a biological basis in the development of the heart instead of it being due to epidemiological variation (21). Beyond the increased prevalence of males having HLHS, there is also significant evidence of sex-based differences in accessibility to surgical care (29). National registry data clearly show that being born female is an independent predictor of infants with HLHS receiving non-operative management, which is associated with near-universal mortality (22). Whether this outcome reflects parental counselling, variation across institutions, or other factors requires further investigation (27, 28). Variations in healthcare-seeking behaviour and adherence to long-term follow-ups between sexes may play a role. In congenital heart disease overall, there are differences between males and females in follow-up attrition, involvement with adult congenital heart disease services, and long-term monitoring, which may lead to variations in late complication rates. It is crucial to recognise that males consistently exhibit higher all-cause mortality rates than females across all age groups in the general population, for both cardiac and non-cardiac reasons. Since CDC WONDER data records the underlying cause of death as noted on death certificates, fatalities coded to HLHS (Q23.4) should mainly represent deaths where HLHS was the primary pathological condition. Nonetheless, the role of non-cardiac comorbidities and alternative causes of death in the older age groups (15–24 years) cannot be disregarded. This signifies a significant constraint of this database-level analysis and should be acknowledged when assessing the gender difference in HLHS-related mortality. Contemporary meta-analyses confirm sex-specific differences in postoperative mortality across congenital heart disease phenotypes (23), further supporting the view that the observed disparity reflects a combination of biological, surgical access, and care pathway factors.

### 4.3 COVID-19 era and the post-pandemic rebound

The lowest number of deaths during the study period was recorded in 2020 (*n* = 287), coinciding with the onset of the COVID-19 pandemic. The observed reduction in deaths in 2020 is likely attributable to multiple factors. Reports indicate that elective and semi-elective congenital cardiac surgery was substantially reduced during the early pandemic period to preserve hospital capacity and minimise infection risk (24, 26). In the youngest patients (those under one year old), postponement of scheduled staged palliative procedures—such as the Stage II Glenn and Stage III Fontan operations—might have led to a decrease in perioperative deaths in 2020 for this age group. It is important to note that this mechanism primarily pertains to the youngest patients undergoing initial or early palliation and is less applicable to mortality changes in older adolescents and young adults, in whom established Fontan physiology, rather than ongoing surgical interventions, prevails in the clinical setting.

This analysis reveals a concerning post-pandemic increase in HLHS-related mortality, rising from 290 deaths in 2021 to 358 in 2024, a 24.7% increase from the nadir. Multiple hypotheses may explain this trend, including deferred staged repairs presenting with higher-risk complications in subsequent years; cohorts of children born during the pandemic now reaching the age of surgical palliation; and disruptions to long-term follow-up care leading to late Fontan-related complications. A similar post-pandemic pattern of increased congenital heart disease mortality has been reported in Germany (25). Nonetheless, the existing dataset does not permit causal inference, and the exact mechanisms underlying this rebound cannot be established solely from mortality data. These findings highlight the necessity of ongoing population-level tracking of HLHS outcomes in the post-pandemic period.

## 5 Limitations

Several limitations of this study should be acknowledged. First, ICD-10 death certificate coding (code Q23.4) may not capture the full anatomical spectrum of HLHS, and coding accuracy depends on the quality of death certificate completion. Second, the 0–24-year age range, while capturing the vast majority of HLHS deaths, does not account for adult Fontan patients dying beyond age 24, which may result in underestimation of the total mortality burden. Third, the ecological study design precludes causal inference or adjustment for individual-level clinical variables. Clinical data, including stage at time of death, anatomical subtype, comorbidities, and socioeconomic status, were not available. Relatedly, because CDC WONDER provides only aggregate counts and no individual-level records are released, the average age at death cannot be calculated or compared between the pre-2021 and post-2021 periods; such a comparison requires individual-level statistics or clinical registry data, which fall outside the scope of this database. Fourth, CDC WONDER suppressed data for Asian or Pacific Islander and American Indian or Alaska Native racial subgroups in multiple study years due to low cell counts, precluding race-stratified trend analysis. Fifth, trend analyses, which are based on age groups (e.g., mortality, which was stratified into 4 age strata corresponding to stages of palliation), weren't feasible because CDC WONDER suppresses data at the age-group level in multiple years. Sixth, as this is a mortality database, the total number of patients with HLHS still alive in the US can't be understood from CDC WONDER alone, as the database captures death but fails to capture the rate of incidence and prevalence. Seventh, CDC WONDER fails to give us more information about the underlying cause of death, beyond the ICD-10 code, hence deaths among males with HLHS but due to other non—cardiogenic causes can't be distinguished from the cardiogenic causes, which causes significant hindrance in interpreting the sex disparity in mortality rates. Finally, the 2024 data is preliminary and can be changed at any time by the CDC. Given how complex the Joinpoint regression methodology is, an independent review by a qualified biostatistician is required before any definitive clinical or policy conclusions are drawn from the findings.

## 6 Conclusion

HLHS-related mortality among individuals aged 0 to 24 years declined significantly in the United States from 1999 to 2021 (APC: −2.22%; *p* = 0.006), consistent with advances in staged surgical palliation. Females demonstrated a consistent and significant decline throughout the 26-year study period, whereas males maintained persistently higher age-adjusted mortality rates in both the pre- and post-COVID eras. The exact mechanisms underlying this sex difference are not fully understood and need further investigation. Total HLHS-related mortality decreased to its lowest level in 2020, and then increased consecutively through 2024, a post-pandemic rebound not previously described in the HLHS literature. The causes of this rebound are not determinable from this dataset and represent one of several possible interpretations. Such observations highlight the necessity of ongoing monitoring of patients with HLHS in the post-pandemic period and the advantage of continuous availability of congenital heart disease-specific services.

## Data Availability

The datasets presented in this study can be found in online repositories. The names of the repository/repositories and accession number(s) can be found in the article/Supplementary Material.
